# Protocol for calcium imaging of dorsal and ventral CA1 neurons in head-fixed mice

**DOI:** 10.1016/j.xpro.2023.102439

**Published:** 2023-07-09

**Authors:** Miru Yun, Jonghan Shin, Min Whan Jung

**Affiliations:** 1 Department of Biological Sciences, Korea Advanced Institute of Science and Technology, Daejeon 34141, Korea; 2 Center for Synaptic Brain Dysfunctions, Institute for Basic Science, Daejeon 34141, Korea

**Keywords:** Microscopy, Neuroscience, Cognitive Neuroscience, Molecular/Chemical Probes

## Abstract

In contrast to other techniques utilized in physiological studies, calcium imaging can visualize target neurons located deep in the brain. Here, we present a protocol for one-photon calcium imaging of dorsal and ventral CA1 neurons in head-fixed mice. We describe procedures for injecting GCaMP6f virus, implanting a gradient-index **(**GRIN) lens, and installing a baseplate for Inscopix microscope mounting.

For complete details on the use and execution of this protocol, please refer to Yun et al.^1^

## Highlights

• One-photon calcium imaging of dorsal and ventral CA1 neurons in head-fixed mice
• GCaMP virus injection, tissue removal, and GRIN lens implantation for dorsal CA1 imaging
• GCaMP virus injection and GRIN lens implantation for ventral CA1 imaging
• Installation of a baseplate for Inscopix microscope mounting

## Abstract (editor highlights)

Publisher’s note: Undertaking any experimental protocol requires adherence to local institutional guidelines for laboratory safety and ethics.

## Graphical abstract

**Figure:**
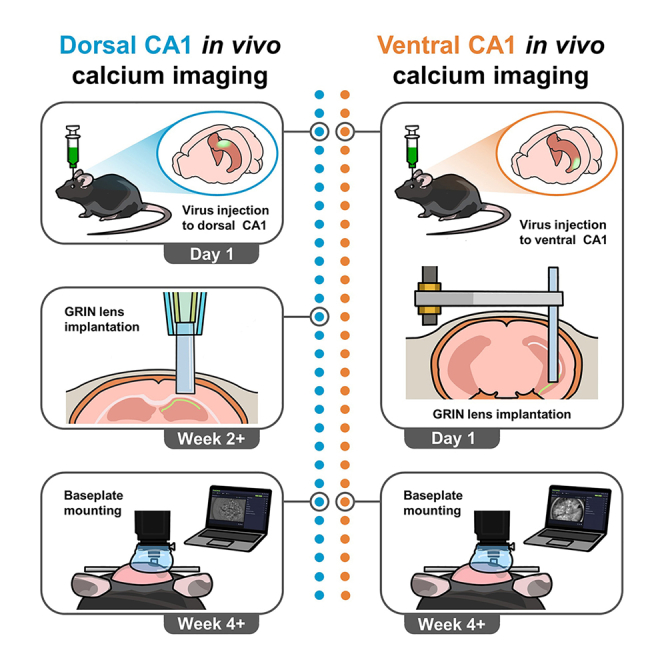

## Before you begin

The majority of physiological studies in rodents have targeted the dorsal, rather than ventral, hippocampus because the latter region is located deep in the brain, making it difficult to identify the cell body layer with an electrode. Calcium imaging, which enables visualization of target neurons, alleviates this problem. We recently compared neuronal activity in the dorsal and ventral hippocampus using one-photon calcium imaging.^1^ The following protocol describes procedures for imaging calcium signals from pyramidal cells expressing GCaMP6f proteins^2^ in the dorsal and ventral CA1 regions. The protocol’s main goals are to maximize cell yield and field of view stability. Drawings of the head plate used for head fixation as well as two different lens holders used to implant a GRIN lens in the dorsal and ventral CA1 are included.

### Institutional permissions

The experimental protocol was approved by the Committee on Animal Research at Korea Advanced Institute of Science and Technology (KAIST; approval number 2020-10). Mice were housed in the mouse facility at KAIST and maintained according to the Animal Research Requirements of KAIST.

### Surgical instruments

**Timing: 10 min**

1. Prepare the following items before surgery: Eye lubricant, a stainless steel bowl, saline, 3M Vetbond, lidocaine, ketoprofen, chlorhexidine gluconate solution, Kwik-Cast, Kwik-Sil, forceps, surgical scissors, a drill bit (tip diameter: 0.5 mm), a 1 mL syringe, an insulin syringe, a 26 gauge needle, 5-0 monocryl suture, Cutanplast, and cotton swabs (Figure 1).

**Figure 1:**
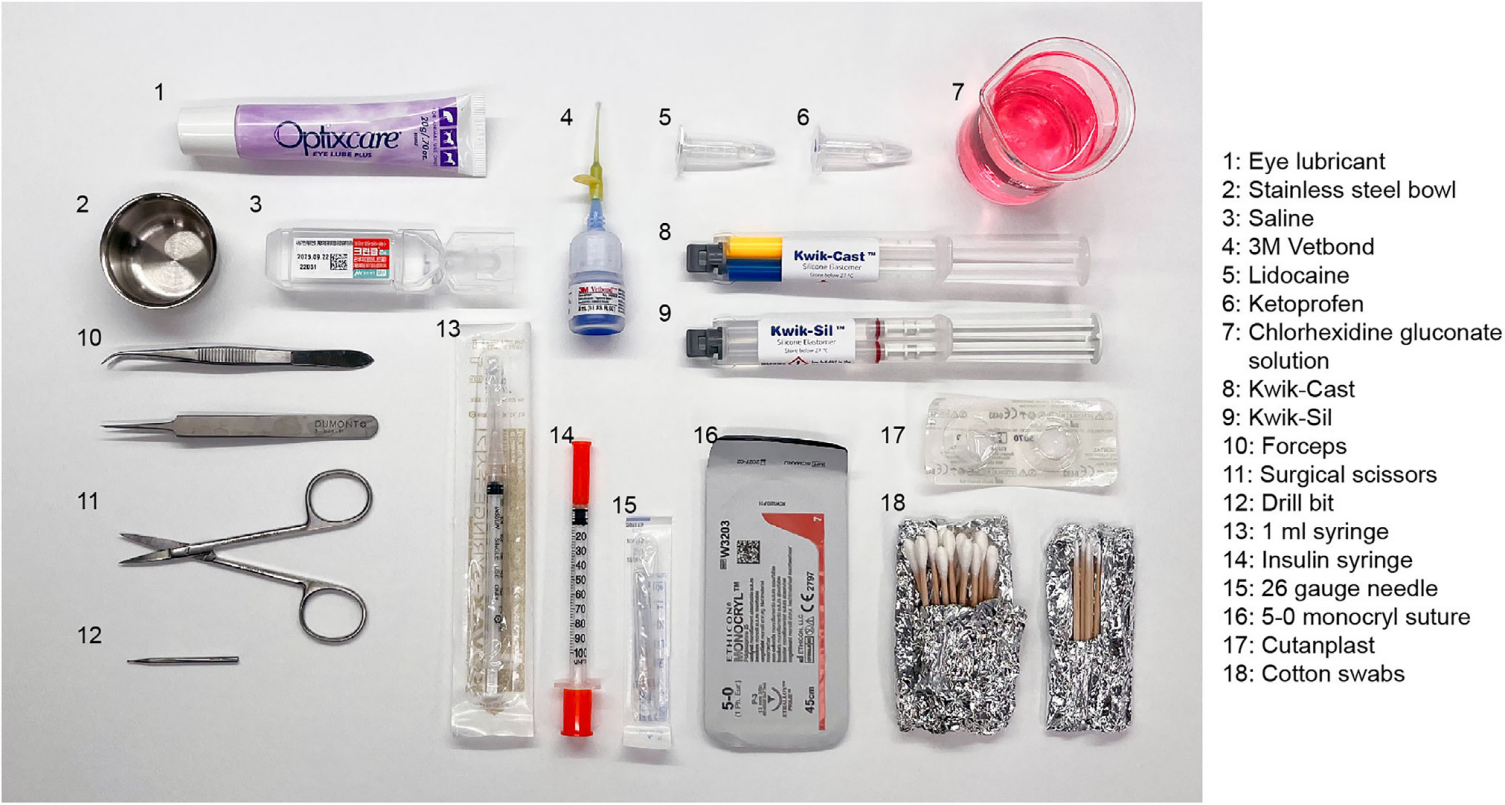
The surgical instruments
2. Sterilize surgical instruments.
  a. Use an autoclave to sterilize surgical scissors, forceps, and cotton swabs.
  b. Surgical instruments must be soaked in chlorhexidine gluconate solution when not in use.

### Virus dilution

**Timing: 30 min**

3. Prepare the following items: a pipette, the viral stock (AAV1-CamKII-GCaMP6f-WPRE-SV40), an Eppendorf tube, and saline solution.

***Note:*** Viral stock concentration varies across lots.

4. Dilute the viral stock with saline to a 2 × 10^12^ vg/ml virus solution in an Eppendorf tube.
5. Dispense 3 μL each of the diluted virus solution into PCR tubes.
6. Store the diluted virus solution at −83°C in a deep freezer.

### Glass pipette for virus injection

**Timing: 10 min**

7. Prepare the following items: borosilicate glass capillaries (outer diameter: 1.0 mm, inner diameter: 0.75 mm), a vertical micropipette puller, and surgical scissors.
8. Load a glass capillary onto the micropipette puller.
9. Pull each capillary with the following settings: Temperature = 62°C, weight = 'heavy 1′, and pull position adjustment plate = 5 mm.
10. Cut the tip of each glass pipette using the surgical scissors.

***Note:*** The diameter of the glass pipette should be less than 0.0035 mm, and the thin segment of the glass pipette should be longer than 1 cm after cutting.

### Virus injection setup

**Timing: 30 min**

11. Prepare the following items for the virus injection setup: the glass pipette prepared in step 10, a Hamilton syringe, Hamilton RN compression fitting, mineral oil, and syringe pump (Figure 2).

**Figure 2:**
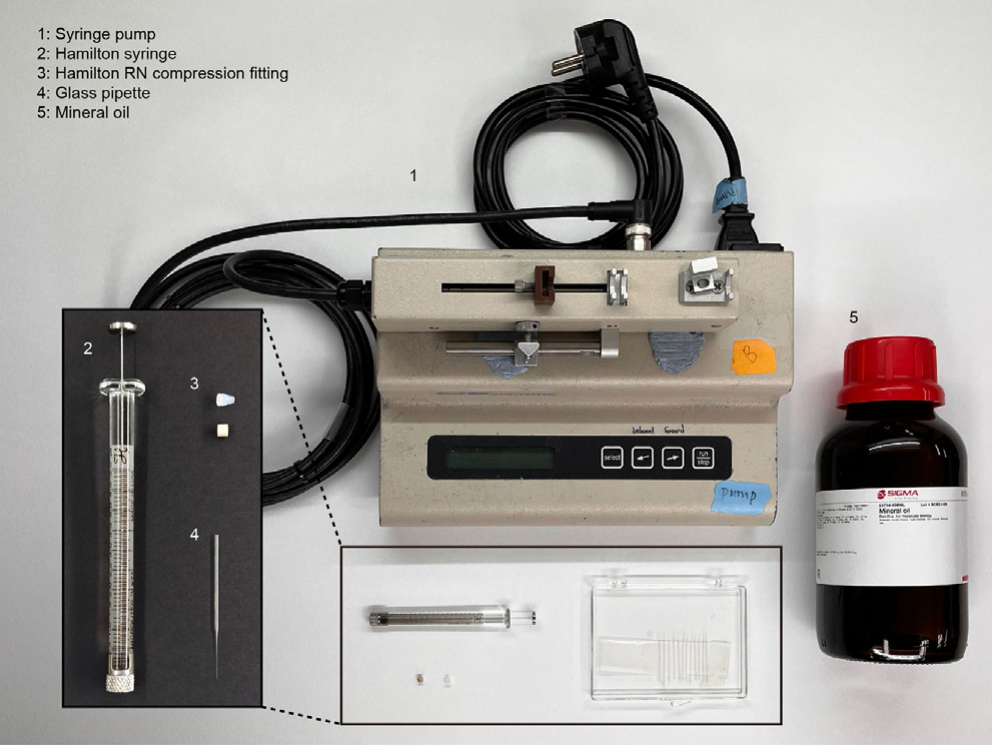
The virus injection setup
12. Connect the Hamilton syringe and the glass pipette together using the Hamilton RN compression fitting.
13. Fill the Hamilton syringe with mineral oil.
14. Load the Hamilton syringe on the syringe pump.

### Suction setup

**Timing: 10 min**

15. Prepare the following items: A suction pump with a maximum pressure of greater than 50 kPa, an 8 mm diameter hose, a 1 mL syringe, and a 24 gauge needle (Figure 3).

**Figure 3:**
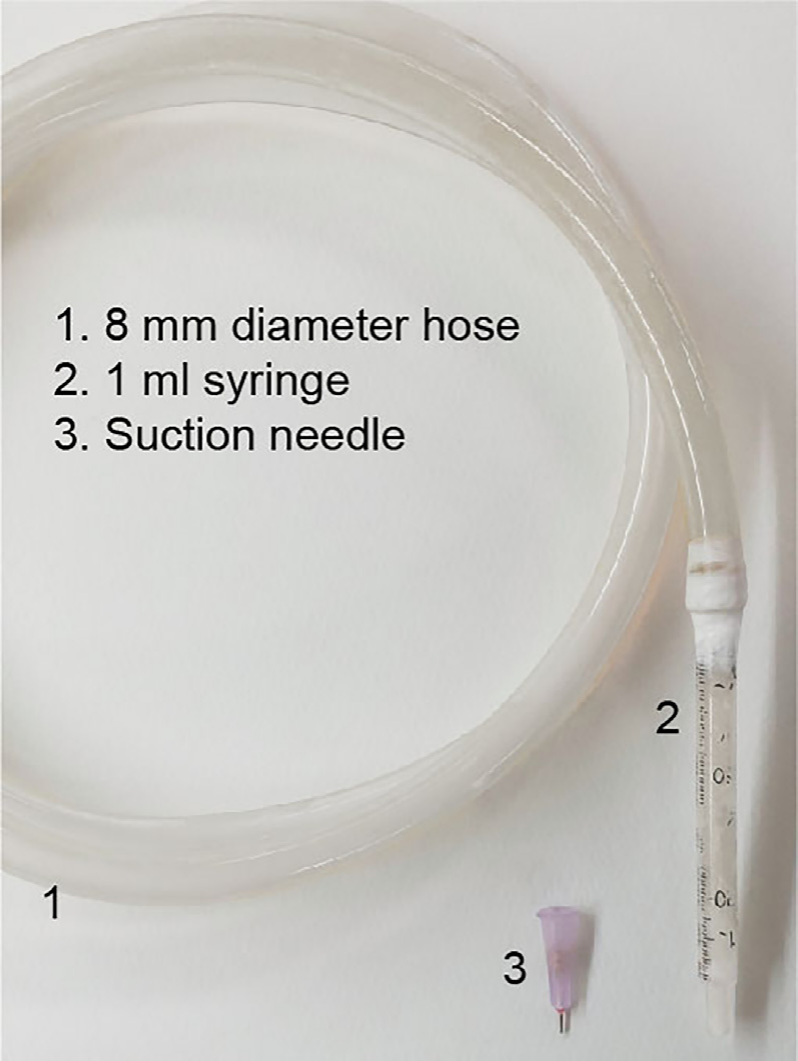
Components of the suction setup
16. Make a suction needle by cutting the 24 gauge needle and creating a flat end.
17. Assemble the suction setup.
  a. Connect one end of the hose to the suction pump and the other end to the syringe barrel.

***Optional:*** Use Teflon tape to create airtight seals on any joints through which air escapes. Connect the suction needle in step 16 to the syringe.

### Dorsal lens holder

**Timing: 20 min**

18. Prepare the following items: A suction pump with a maximum pressure of greater than 50 kPa, a 4 mm-diameter hose, a 1 mL syringe, a stereotaxic holder, two P1000 tips, one P200 tip, instant glue (Loctite 401 or steel epoxy), labeling tape, and a razor blade.
19. Cut one P200 tip and one P1000 tip with a razor blade as follows (Figure 4A):
  a. Cut the P200 tip at 24.5, 27 and 45 mm from the narrow tip end. Use the 0–24.5 and 27–45 mm segments.
  b. Cut the P1000 tip at 2 and 27 mm from the narrow tip end. Use the 2–27 mm segment.

**Figure 4:**
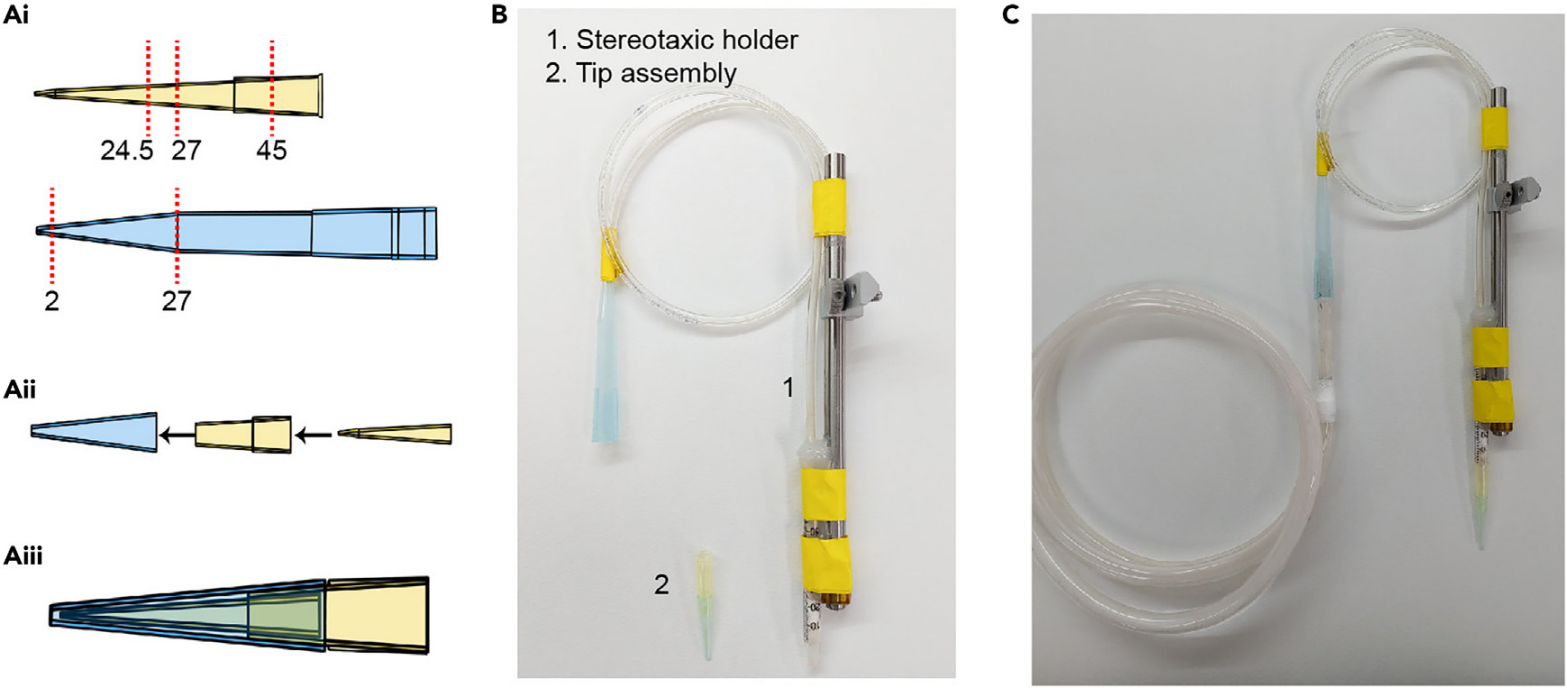
The dorsal lens holder (A) Diagram of dorsal lens holder preparation. Cut the P200 and P1000 tips (unit: mm) along the vertical dashed lines shown in Ai, and assemble the tip segments as shown in Aii and Aiii. (B) Components of the dorsal lens holder. (C) The lens holder is connected to the suction setup.
20. Insert the P200 0–24.5 mm segment into the P200 27–45 mm segment and secure it with glue (Figure 4A).
21. Cover the narrow end of the glued P200 tip segments with the P1000 tip segment and secure it with glue. Note that the P200 and P1000 tip ends should be 1 mm apart (Figure 4A).
22. Assemble the dorsal lens holder (Figure 4B).
  a. Connect one side of the 4 mm diameter hose to the narrow end of an intact blue tip and the other side to the syringe barrel.
  b. Attach a 1 mm syringe parallel to the stereotaxic holder with glue and labeling tape.
    ***Optional:*** Use Teflon tape to create airtight seals on any joints through which air escapes.
  c. Connect the P1000 tip to the suction setup’s syringe described in step 17 (Figure 4C).
  d. Connect the tip assembly in step 21 to the syringe in step 22b.

### Ventral lens holder

**Timing: 5 min**

**Figure 5:**
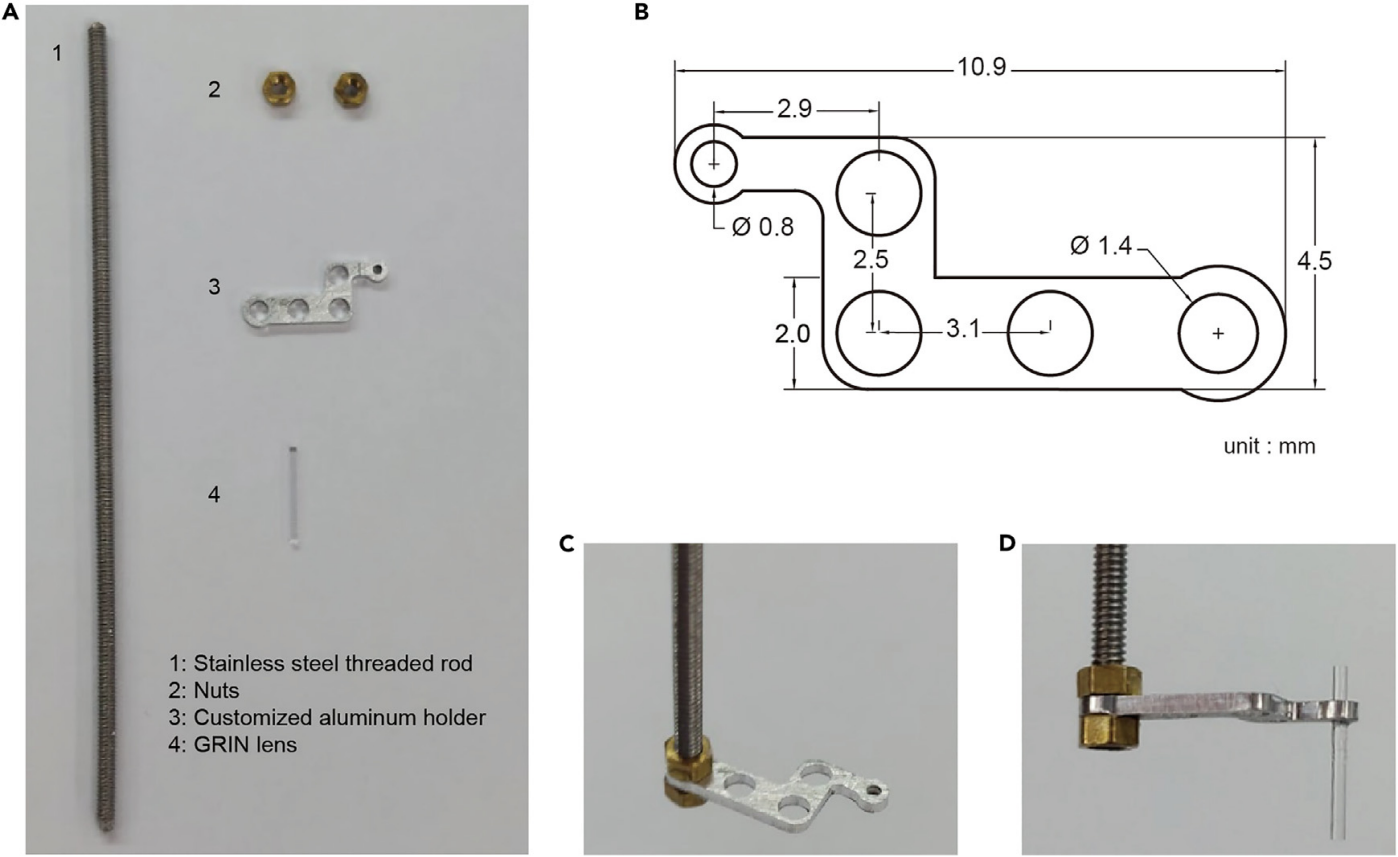
The lens holder for ventral lens implantation (A) The components of the lens holder. (B) Diagram of the customized aluminum holder (unit: mm). (C) Two nuts secure the aluminum holder to the rod. (D) The lens is glued to the smallest hole of the holder.

***Note:*** The size of a GRIN lens for the ventral CA1 differs from that for the dorsal CA1.

23. Prepare the following items: Stainless steel threaded rod (M1.4 × 40–60 mm), two nuts (M1.4 × 1 mm), a GRIN lens (0.6 × 7.3 mm), a customized 1 mm thick aluminum holder, and glue (Loctite 401 and Kwik-Sil) (Figure 5A).

24. Use two nuts to secure the aluminum holder to the rod (Figure 5C).

***Optional:*** Wrap the nuts and holder with Kwik-Sil for a secure fit.

25. Insert the lens vertically into the holder’s smallest hole, allowing at least 5 mm of the lens’s bottom to protrude. (Figure 5D).
26. Apply glue (Loctite 401) between the lens and the lens holder using a 24 gauge needle.

### Head plate

**Timing: 5 min**

**Figure 6:**
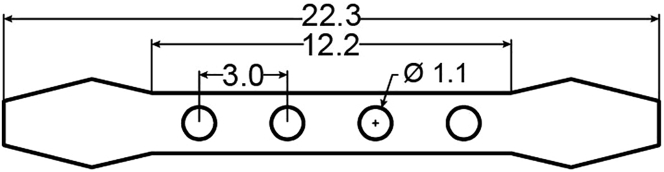
Diagram of the customized aluminum head plate (unit: mm)

27. Prepare a 1 mm thick customized aluminum head holder (Figure 6).

## Key resources table

**Table:** 

REAGENT or RESOURCE	SOURCE	IDENTIFIER
**Bacterial and virus strains**		

AAV1-CamKII-GCaMP6f-WPRE-SV40	Penn Vector Core	100834-AAV1

**Chemicals, peptides, and recombinant proteins**		

Saline solution	JW Pharmaceutical Corporation	N/A
Lidocaine HCl Hydrate Injection 2%	Daihan	N/A
Bupivacaine HCl Heavy Injection	Myungmoon	N/A
Ketoprofen Injection 100 mg/2 mL	Bukwang	N/A
Chlorhexidine gluconate solution 5%	Sungkwang	N/A
Mineral oil	Sigma Aldrich	69794
Isoflurane (Terrell)	Piramal	NDC 66794-011-25

**Experimental models: Organisms/strains**		

Mouse: C57BL/6J (2–6 month, male or female)	Jackson Laboratory	JAX000664; RRID:MGI:2160085

**Software and algorithms**		

nVista Data Acquisition Software	Inscopix	1.3–1.6
ImageJ	NIH	N/A

**Other**		

Eye lubricant (Optixcare Pet Eye Lube Plus)	Aventix Animal Health	OPX-4242
3M Vetbond	3M Healthcare	1469sb
Kwik-Cast	World Precision Instruments	KWIK-CAST
Kwik-Sil	World Precision Instruments	KWIK-SIL
Forceps	Fine Science Tools	11254-20, 11051-10
Surgical scissors	Fine Science Tools	14060-10
Drill bit (tip diameter: 0.5 mm)	Fine Science Tools	19007-05
5-0, P-3, monocryl suture	Ethicon	W3203
Cutanplast (dental)	Mascia Brunelli	05610101
Borosilicate glass capillaries	World Precision Instruments	TW100-4
Vertical micropipette puller	Narishige	PC-10
Hamilton syringe (75RN, no needle)	Hamilton Company	7634-01
Hamilton RN compression fitting	Hamilton Company	55750-01
Syringe pump	KD Scientific	Model: 310 plus
Suction pump	Cliq	VC-701
8 mm (outer diameter) hose	Tygon	e-3603
4 mm (outer diameter) polyurethane straight hose	Sang-A	N/A
Stereotaxic holder (standard electrode holder)	KOPF	Model 1770
GRIN lens (1.0 × 4.0 mm)	Inscopix	1050-004595
GRIN lens (0.6 × 7.3 mm)	Inscopix	1050-004597
Homeothermic system for controlling body temperature in small animals	Harvard Apparatus	Model: HB101
Self-tapping screws (Phillips head, M1 × 3.0 mm)	N/A	N/A
Super Bond C&B (L-type radiopaque)	Sun Medical	0334-0020
Super Bond C&B (Monomer)	Sun Medical	0334-0013
Super Bond C&B (Catalyst)	Sun Medical	0334-0010
Dental cement	Vertex	Type 2, Class 2
Inscopix microscope	Inscopix	nVista 3.0
Baseplate	Inscopix	1050-004638
Baseplate cover	Inscopix	1050-004639
Black cement (Contemporary Ortho-Jet Liquid)	Lang Dental Mfg. Co.	1506, black
Black cement (Contemporary Ortho-Jet Powder)	Lang Dental Mfg. Co.	1530, black
UV glue (Norland Optical Adhesive)	Norland Products	NOA 63
Nail polish (Black)	Wakemake	11

## Materials and equipment

Drug concentrations may differ between brands. Dilute as necessary.

• Lidocaine solution: Mix 2.5 mL of 2% lidocaine hydrochloride hydrate (21.33 mg/mL) and 2.5 mL of bupivacaine (5 mg/mL), dilute the mixture with 5 mL of saline at 25°C, aliquot it into 0.5 mL volumes, and store at 4°C.
• Ketoprofen solution: Mix 2 mL of ketoprofen (50 mg/mL) and 98 mL of saline at 25°C, aliquot it into 0.5 mL volumes, and store at 4°C.
• Chlorhexidine gluconate solution: Mix 100 mL of chlorhexidine gluconate solution (0.25 mL/mL) and 900 mL of 70% ethanol at 25°C, and store at 4°C.

## Step-by-step method details

### Virus injection in dorsal CA1

**Timing: 2–3 h**

Calcium imaging requires both viral expression and GRIN lens insertion in the target location. For dorsal CA1 calcium imaging, we separate the virus injection and lens implantation steps because performing both steps concurrently may lead to viral elimination due to aspiration of brain tissue for lens implantation. Therefore, we inject the virus and wait for adequate viral expression before implanting the lens. Setting the precise surgical coordinate is a critical step in this process.

1. Anesthetize the mouse.
  a. Put the mouse in an anesthesia induction chamber and fill it with 3% isoflurane at a rate of 1 L/min for anesthesia by inhalation.

***Note:*** This step takes about 5 min. The criteria for successful anesthesia are relaxation of the body and tail muscles, gasping once every second, and no response to toe or tail pinching.

2. Fix the mouse’s head in the stereotaxic.
  a. Place the mouse’s nose inside an anesthesia mask supplying 3% isoflurane.
  b. Fix the mouse’s head with ear bars.
  c. Set the Homeothermic system temperature to 33.5°C to keep the mouse body temperature stable during surgery.
3. Trim the hair and incise the skin on top of the head (Figure 7).
  a. Disinfect the surgical site with chlorhexidine gluconate solution.
  b. Use scissors to trim the hair.
  c. Clean the surgical site with chlorhexidine gluconate solution.
  d. Dab the surgical site with lidocaine solution (∼100 μL).
  e. Inject ketoprofen solution via IM (100 μL/g).
  f. Make a 1.2 cm incision along the midline of the skin (Figure 7).
  g. Apply Eye lubricant to the mouse’s eyes to keep them from drying out.

**Figure 7:**
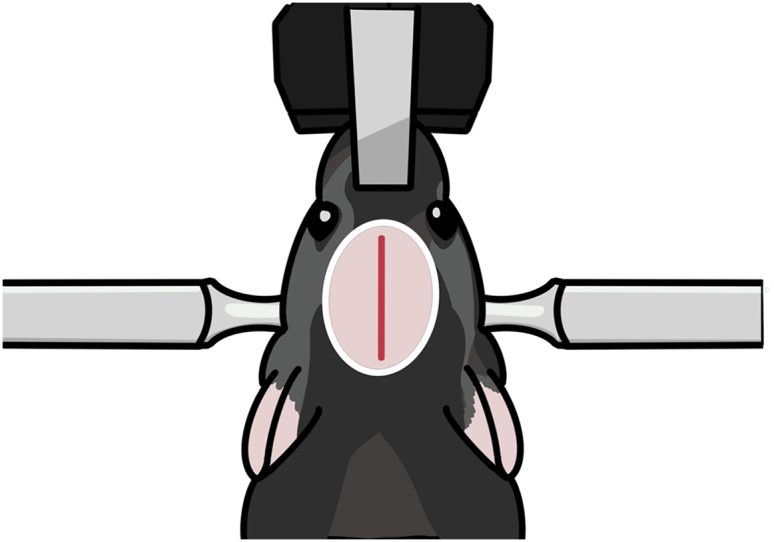
Diagram of the surgical area The pink oval indicates the hair-trimming area. The red vertical line indicates the incision line.
4. Scrub the skull.
  a. Gently push the skin to the left and right with sterilized cotton swabs to widen the incision.
  b. Clean the exposed skull with a sterilized dry cotton swab and a saline-dipped cotton swab. Troubleshooting 5.
5. Level the skull.
  a. Fasten a 22–26 gauge needle to the stereotaxic holder.
  b. Put ink on the tip of the needle and gently touch the bregma and lambda with the needle to mark them.
    ***Note:*** The bregma is the point at which the trend line of the coronal suture meets the midline, and the lambda is the center of the triangle whose vertices are the intersection point of the lambdoid suture with the sagittal suture and the bent points of the lambdoid suture.
  c. Estimate the difference between the bregma and lambda along the dorsal-ventral axis using the needle.
  d. Level the bregma and lambda along the dorsal-ventral axis by adjusting the dorsal/ventral adjustment dial of the stereotaxic.
  e. Repeat steps 5c–d until the relative difference between the bregma and lambda is smaller than 0.02 mm.
6. Mark the surgical site (Figure 8A).
  a. Mark the point 1.94 mm posterior to the bregma.
  b. Mark the target point 1.40 mm laterally from the point marked in 6a.
  c. Make four marks, one each in the anterior, posterior, medial, and lateral directions, 0.60 mm from the target point.

**Figure 8:**
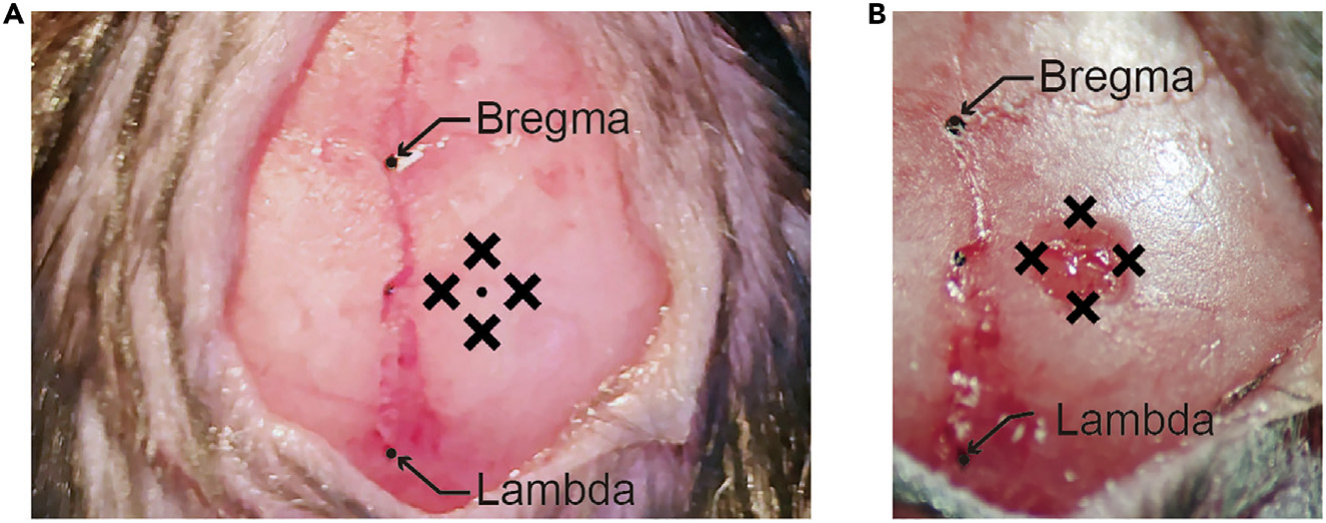
Skull marking and drilling for dorsal CA1 virus injection (A) Marking the drilling site. (B) A square-shaped hole was made by drilling.
7. Drill a hole (Figure 8B).
  a. Mount the drill bit on a micro hand drill.
  b. Create a square-shaped opening in the skull by drilling (the length of sides: ∼1.2 mm).
    i. Drill the largest hole possible within the area marked in step 6c.
    ii. Verify that the lens fits into the opening.

***Note:*** It is recommended to take care not to tear the dura mater.

8. Inject virus.
  a. Prepare virus injection.
    i. Fill the Hamilton syringe with the virus (1 μL for each mouse).
  b. Locate the injection site.
    i. Place the Hamilton syringe over one of the target sites (site 1, 1.94 mm posterior and 1.15 mm lateral to the bregma; site 2, 1.94 mm posterior and 1.6 mm lateral to the bregma).
    ii. Lower the Hamilton syringe to 1.1 mm below the brain surface, which is 0.05 mm above the target site.
      **Pause point:** Wait 5 min for the brain tissue to stabilize.
  c. Inject 0.4 μL of the virus at a rate of 0.05 μL/min. It takes 8 min to inject the virus.
    i. Verify that the virus level in the Hamilton syringe decreases.
      ***Note:*** If the virus level does not decrease, remove the Hamilton syringe from the brain and gently clear the tip of the glass pipet with saline-soaked Cutanplast. If it doesn’t work, then cut the tip of the glass pipet slightly or replace it with a new one.
      **Pause point:** Allow 10 min after virus injection for the virus to spread in the brain tissue.
  d. Move the Hamilton syringe upwards.
  e. Repeat steps 8b–8f for the injection at the second site.
9. Seal the hole.
  a. Blend the Kwik-Sil solution.
  b. Fill the hole with the Kwik-Sil mixture.

**Pause point:** Wait 3 min for the mixture to set.

10. Suture the skin.
  a. Insert a suture needle through the skin on both sides.
  b. Tie a knot.
  c. Repeat steps 10a and 10b until the skin is completely closed.

**Pause point:** Wait for 2 weeks for viral expression. Viral injection and lens implantation are performed on two different days as explained earlier.

### Lens implantation in the dorsal CA1

**Timing: 1–2 h**

The brain tissue above the target recording site is removed, a GRIN lens (1.0 × 4.0 mm) is positioned in the target site, and the lens is fixed with cement. Properly positioning the lens and securely fixing it in place are critical steps here.

**Figure 9:**
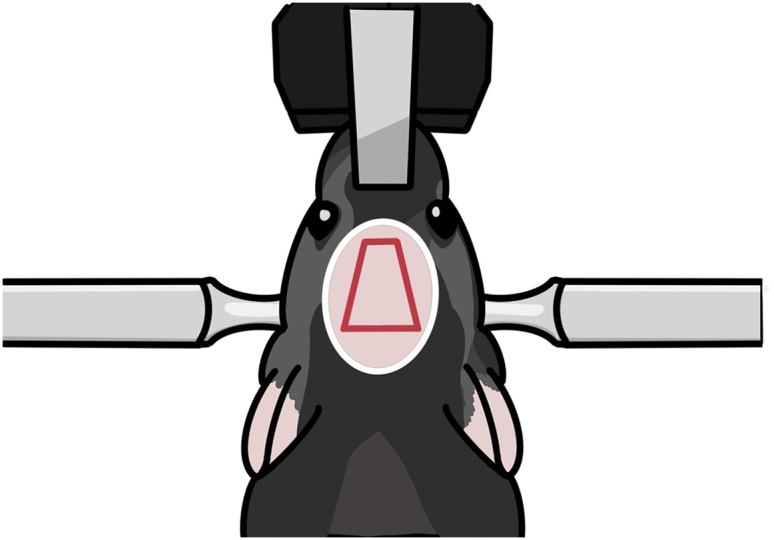
Diagram for skin incision The pink oval indicates the hair-trimming area. The red quadrangle indicates the incision area.
  b. Apply 3M Vetbond along the cutting line of the skin to attach the skin to the skull. Troubleshooting 2.

***Note:*** Skipping step 11b may lead to skin inflammation; it may also allow skin growth into the space between the skull and cement, ultimately causing the cement to detach from the skull.

**Figure 10:**
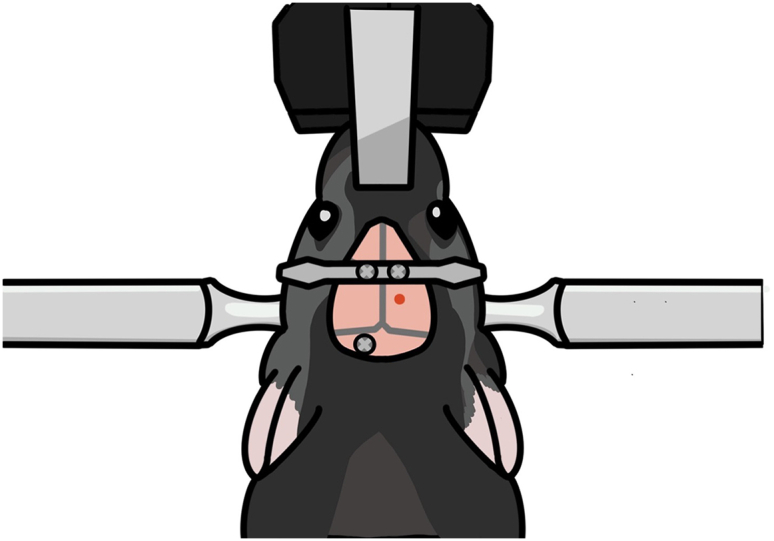
Diagram for fixing the head plate and screws on the skull The head plate is secured with two screws slightly in front of the coronal suture. An additional screw is secured to the skull above the cerebellum.

***Note:*** Fasten the screws to barely penetrate the skull (less than 1 mm deep from the skull surface). Avoid overtightening the screws, which may damage brain tissues and cause inflammation.

11. Repeat steps 1–5 except for hair trimming and skin incision.
  a. Remove skin with an isosceles trapezoid incision (Figure 9).
    i. Make a small skin incision along the ear bar line about the length of the distance between the eyes.
    ii. Cut the skin obliquely to the inner corner of each eye, leaving enough space between the eyes and incision lines.
    iii. Cut the skin near the eyes.

12. Fix the head plate with screws (Figure 10). Troubleshooting 5.
  a. Soak the head plate and screws in chlorhexidine gluconate solution for sterilization.
  b. Align the head plate slightly in front of the coronal suture and use a micro hand drill to create an indentation on the skull beneath each screw hole.
  c. Mark the location for another screw above the cerebellum and at that location make another indentation on the skull using a micro hand drill.
  d. Use a screwdriver to secure the head plate and the screw in point 12c.

**Figure 11:**
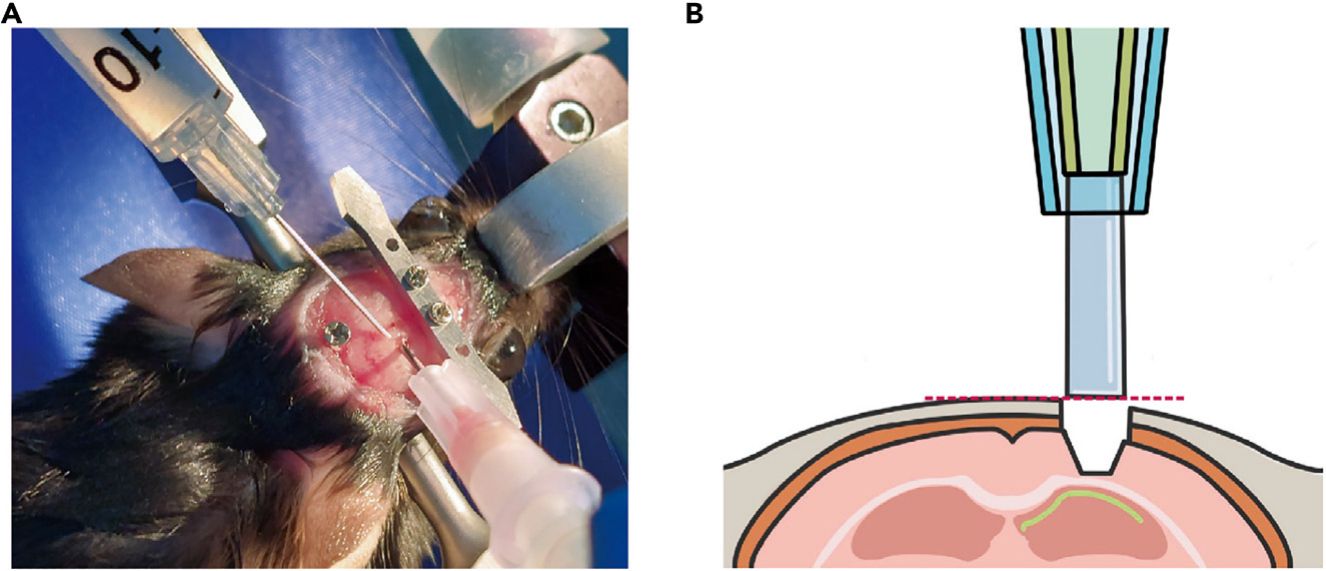
Aspiration of the brain tissue above the dorsal CA1 target site (A) Photograph taken while aspirating. The brain tissue above the hippocampus is removed while saline is supplied. (B) Schematic showing the removal of the brain tissue above the hippocampus and the positioning of the lens. The red dotted line indicates the reference point for lens positioning along the dorsal-ventral axis.

**Figure 12:**
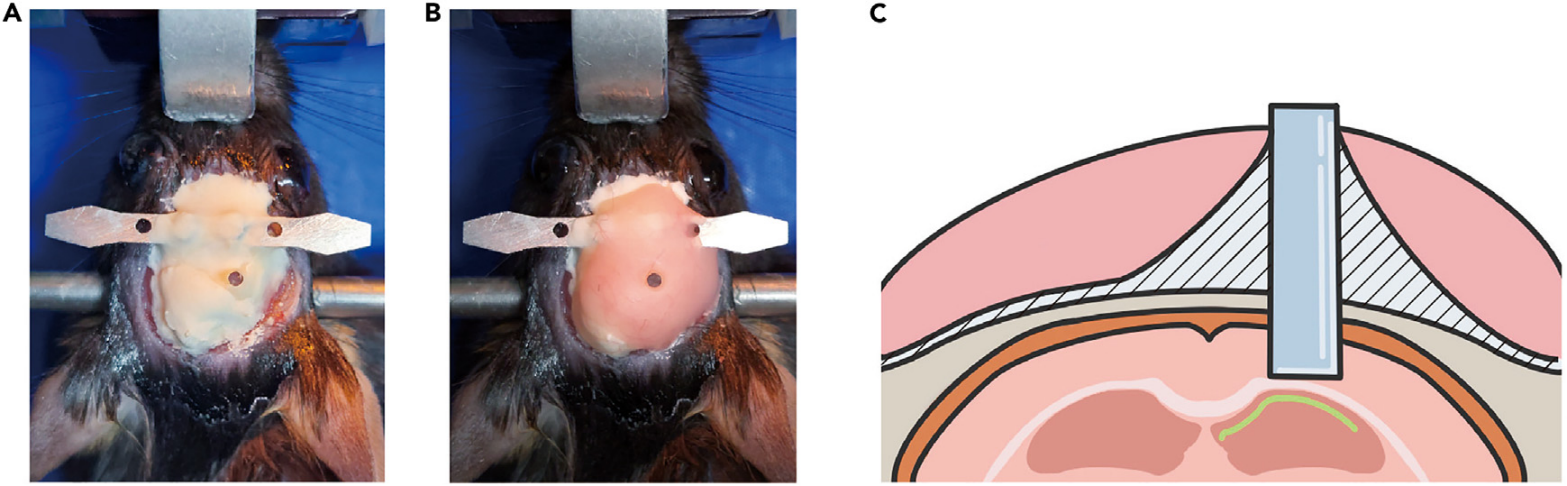
Cementing the lens in place after dorsal CA1 implantation (A) Photograph taken after the initial Super Bond C&B cementing. (B) Photograph taken after the second dental cementing. (C) Diagram showing how the lens is cemented. The lens is fixed first with Super Bond C&B (hatched area) and then with dental cement (pink).

13. Remove the cortex above the hippocampus (Figure 11A).
  a. Remove the Kwik-Sil applied in step 9 to expose the hole.
  b. Set up the suction setup.
  c. Turn on the suction pump and set the vacuum pressure at over 50 kPa.
  d. Remove the cortex over the target site by aspiration until the corpus callosum appears (Figure 11B).
    i. Place the suction needle tip on the brain tissue to be removed. While absorbing, use an insulin syringe to apply saline to ensure a clear view and prevent blockage.
    ii. Remove brain tissue in the middle of the hole more deeply than at the edge, creating a funnel-shaped hollow by adjusting the angle of the suction needle.
    iii. Stop the aspiration when white tissue (corpus callosum) appears (Figure 11B).
      ***Note:*** Make sure not to over-remove brain tissue. The cortical tissue surrounding the hollow plays a role in holding the implanted lens in place. Troubleshooting 3
  e. Turn off the suction pump.
  f. Absorb blood using Cutanplast. Troubleshooting 2.
    i. Fill the hollow with saline-soaked Cutanplast.
      ***Note:*** Hydrate the Cutanplast with saline to prevent the blood clot formation.
    ii. Wait until bleeding stops.
      ***Optional:*** Change the Cutanplast with a new one if it cannot absorb all of the blood.
    iii. After bleeding has stopped, use Cutanplast to gently remove any remaining blood clots.
    iv. If bleeding resumes, apply Cutanplast until it stops.
14. Implant the lens.
  ***Note:*** The GRIN lens (1.0 × 4.0 mm) should be sterilized with chlorhexidine gluconate solution prior to implantation. The size of a GRIN lens for the dorsal CA1 differs from that for the ventral CA1.
  a. Fix the dorsal lens holder to the stereotaxic holder.
  b. Turn on the suction pump and increase the vacuum pressure to more than 50 kPa.
  c. Load the lens onto the tip assembly.
    ***Note:*** Make sure that the lens holder holds the lens firmly in place. Ensure that the lens plane is parallel to the skull.
  d. Align the center of the lens to the reference point in step 6a (1.94 mm posterior and 1.4 mm lateral to the bregma).
  e. Adjust the lens’ dorsal-ventral positioning using the skull as a reference (Figure 11B).
    i. Lower the lens to just above the skull.
    ii. Carefully move the lens to the medial side and confirm that it is just above the skull.
      ***Note:*** The surface of the medial edge of the hole in the skull serves as the dorsal-ventral axis reference point.
  f. Lower the lens to 1.7 mm below the position in step 14e.
    i. Lower the lens to 0.7 mm below the position in step 14e (this can be done rapidly).
    ii. Slowly lower the lens a further 1.0 mm at a speed of 0.2 mm/min.
      **CRITICAL:** Stop lowering the lens if blood begins to leak out of the hollow. Use Cutanplast to absorb any minor bleeding and wait for it to stop. In the event of significant bleeding, lift the lens, clean the lens if it is dirty, absorb the blood with Cutanplast, and wait for the bleeding to stop. Then, begin lowering the lens again.
15. Fix the lens with cement (Figure 12).
  ***Optional:*** If the hole is too large and the cortex is exposed between the lens and the skull, fill the gap with Kwik-Sil.
  a. Cover the lens, skull, head plate, and screw with Super Bond C&B (Figure 12A).
    **CRITICAL:** The tip assembly should not be covered with cement.
  b. After the cement has hardened, switch off the suction pump and disconnect the lens holder from the suction pump to relieve pressure.
  c. Remove the lens from the tip assembly.
    ***Optional:*** If necessary, use additional dental cement to cover exposed portions of the skull, lens, head plate, and screw (Figure 12B).
    **Pause point:** Wait for the cement to harden for at least 10 min.
16. Apply Kwik-Cast and Kwik-Sil to the lens to prevent lens damage.
  a. Cover the lens with Kwik-Cast.
  b. Apply Kwik-Sil to cover the upper portion of the dental cement, including the area where Kwik-Cast has been applied.

**Pause point:** Wait for 1–2 weeks for stabilization.

### Virus injection and lens implantation in the ventral CA1

**Timing: 4–5 h**

This step is for ventral CA1 calcium imaging in a new group of mice. In this section, we inject virus into the ventral CA1, lower the lens into the ventral CA1, and fix the lens with cement in the same manner as for dorsal CA1 calcium imaging. However, due to the ventral CA1’s deep location in the brain, the lens is lowered very slowly and without suctioning brain tissue. Setting the precise surgical coordinate is a critical step here.

17. Repeat step 11 with a new mouse.
18. Mark the surgery site (Figure 13).
  a. Mark the point 3.16 mm posterior to the bregma.
  b. Mark the target point 3.50 mm laterally from the point 18a.
  c. Make four marks, one each in the anterior, posterior, medial, and lateral directions, 0.60 mm from the target point.

**Figure 13:**
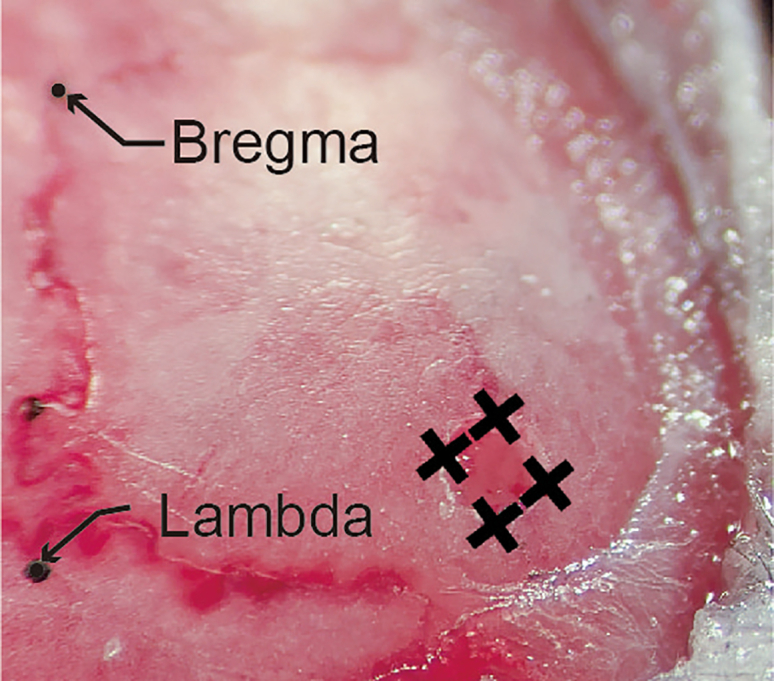
Marking and drilling for ventral CA1 surgery
19. Drill a hole.
  a. Repeat step 7 but with the length of the square-shaped opening’s sides reduced to about 0.8 mm.
20. Inject virus.
  a. Prepare the virus injection as in step 8.
  b. The virus is injected at two sites (3.16 mm posterior and 3.5 mm lateral to the bregma; 3.5 and 3.7 mm ventral to the brain surface).
  c. Each injection has a volume of 0.3 μL, and an injection speed of 0.05 μL/min. It takes about 6 min to complete virus injection.

**Pause point:** Allow 10 min after virus injection for the virus to spread in the brain tissue.

21. Fix the head plate with screws (Figure 14).
  a. Before tightening the screws, make sure that the lens holder, head plate and screws are correctly placed.
    i. The lens holder must be positioned inside the surgical area.
    ii. While being lowered, the lens and the holder should not be obstructed by the head plate.
    iii. The additional screw should be positioned differently than it was during dorsal CA1 surgery to prevent it from colliding with the lens holder.
  b. Repeat step 12.

**Figure 14:**
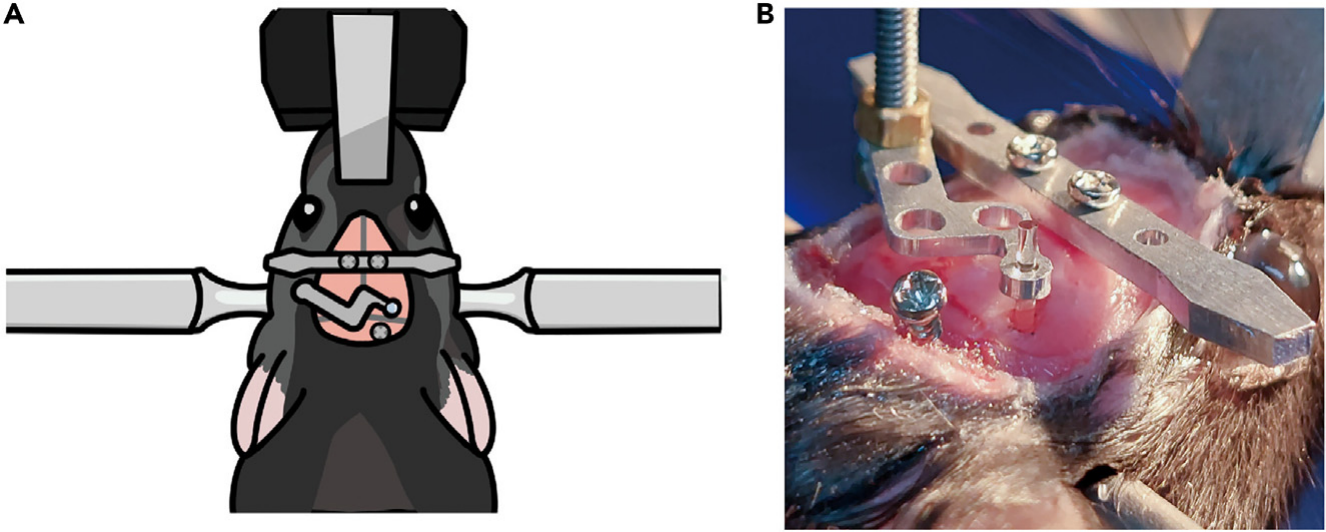
Positioning of the head plate, lens holder, and skull screw (A) Diagram showing the arrangement of the head plate, lens holder, and skull screw. These elements should be positioned far enough apart that they do not obstruct each other. (B) Photograph showing the placement of the head plate, lens holder, and skull screw after lens implantation in the ventral CA1.
22. Locate the lens target site.
  a. Install the lens holder onto the stereotaxic holder.
  b. Adjust the lens position as described in step 14e.
  c. Position the GRIN lens (0.6 × 7.3 mm) to 3.16 mm posterior and 3.5 mm lateral to the bregma.
23. Lower the lens. Troubleshooting 3.
  a. Lower the lens by 0.2 mm every 2 min, down to 2.0 mm from the brain surface.
  b. Lower the lens by 0.1 mm every 2 min, down to 3.0 mm from the brain surface.
  c. Lower the lens by 0.05 mm every 2 min, down to 3.6 mm from the brain surface.

**CRITICAL:** Stop lowering the lens if blood begins to leak out during insertion. Absorb blood with Cutanplast and wait for bleeding to stop. Then, begin lowering the lens again. Troubleshooting 2

24. Fix the lens with cement (Figure 15A).
  a. As in step 15, cover the lens, head plate, and screws with Super bond C&B.
  b. Cover the first and second holes of the lens holder with Super bond C&B, but leave the third hole uncovered.

**Figure 15:**
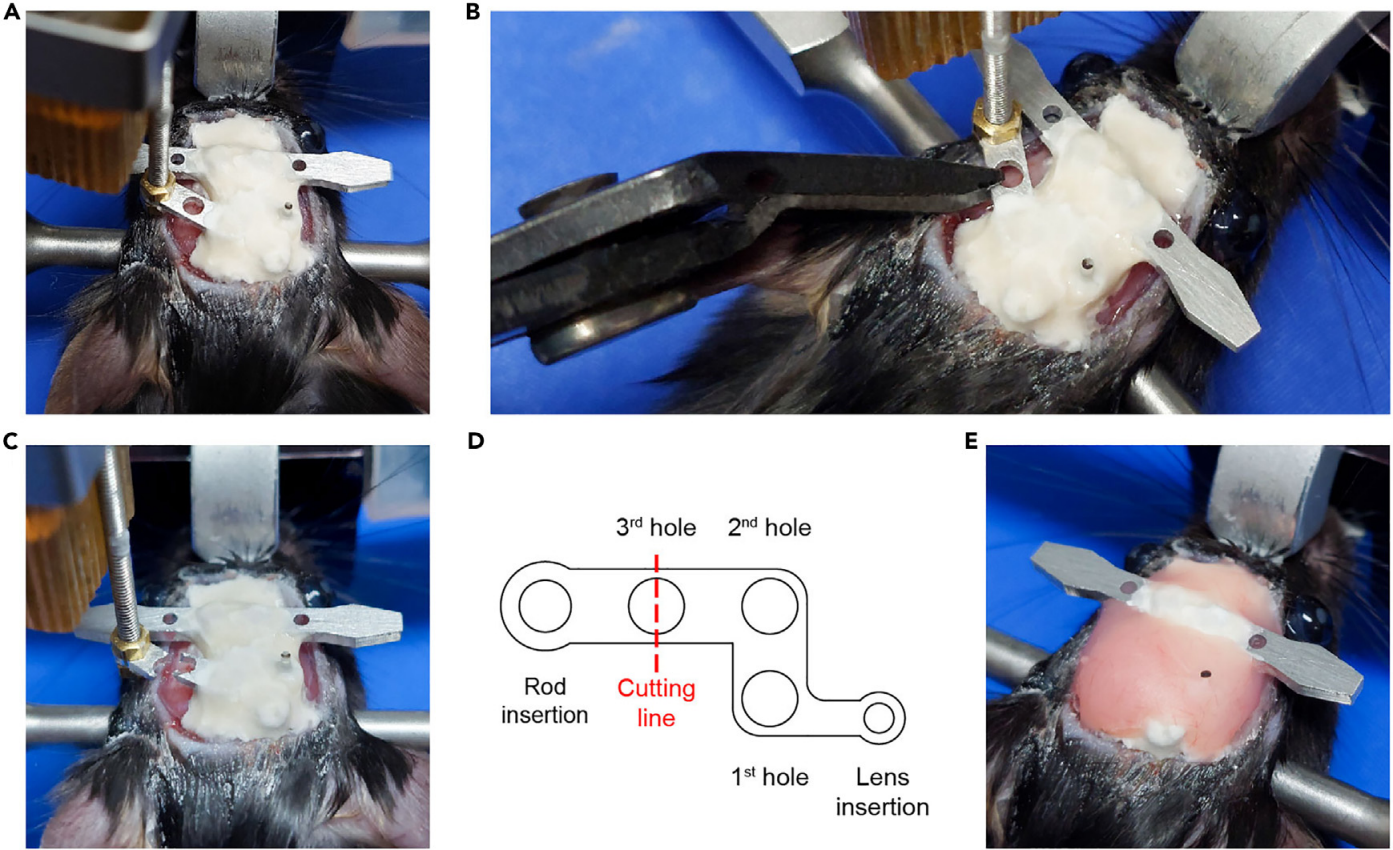
Cementing the lens in place after ventral CA1 implantation (A) Photograph taken after the initial Super Bond C&B cementing. (B) Photograph taken during cutting the lens holder. (C) Photograph taken after cutting the lens holder. (D) Illustration of the cutting line (in red). (E) Photograph taken after the second dental cementing.
25. Disconnect the lens from the holder (Figures 15B–15D).
  a. Gently cut the third hole in the lens holder with nippers (Figures 15B and 15D).

**CRITICAL:** The thickness of the lens holder is 1 mm. Do not cut the lens holder all at once, as the lens may be damaged during cutting. Rather, cut the posterior side of the hole first, and then the anterior side.

26. Apply cement again to the lens.
  a. Apply dental cement to cover the cut lens holder and to make the dental cement surface rounded and smooth.

***Optional:*** If necessary, use additional dental cement to cover exposed portions of the skull, lens, head plate, and screws (Figure 15E).

**Pause point:** Wait for the cement to harden for at least 10 min.

27. Apply Kwik-Cast and Kwik-Sil to the lens to prevent lens damage.

**Pause point:** Wait for 3–4 weeks for viral expression.

### Baseplate mounting

**Timing: 1–2 h**

The Inscopix microscope (nVista) is mounted on the baseplate. In this step, a UV resin is used to build a baseplate foundation and fix the baseplate to the foundation. The mounting procedure is the same for the dorsal and ventral CA1. Proper positioning of the baseplate is critical because the microscope’s focus range is restricted.

**Figure 16:**
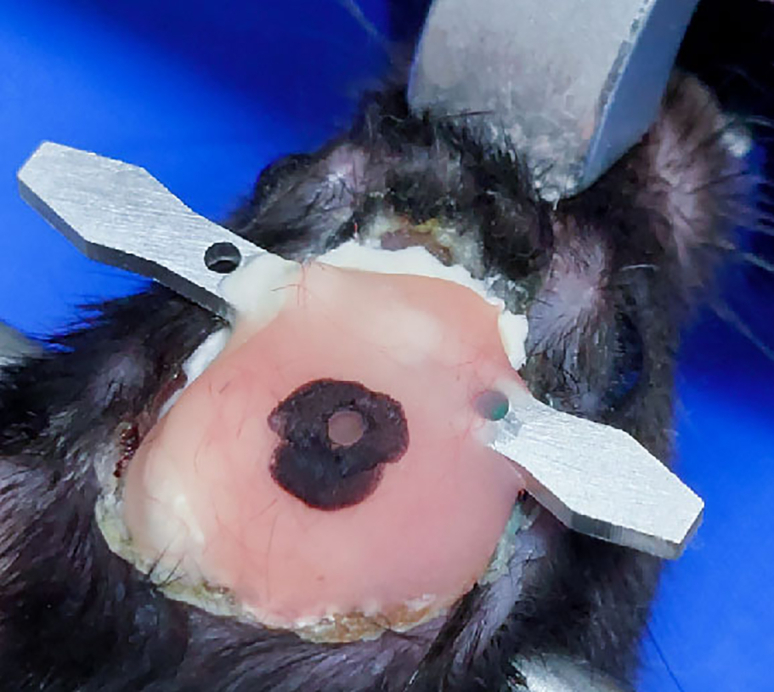
Surrounding the lens with black cement

28. Repeat steps 1–2.
29. Remove the Kwik-Sil and Kwik-Cast cover and clean the lens.
  a. Remove the Kwik-Cast and Kwik-Sil using forceps.
  b. Gently wipe the lens with a cotton swab soaked with 70% ethanol.
30. Find the focal plane.
  a. Set up the nVista system.
  b. Assemble the baseplate and the Inscopix microscope.
    ***Note:*** A loose connection between the microscope and baseplate may cause a change in focal plane after fixing the microscope-baseplate assembly.
  c. Place the microscope above the lens.
  d. Turn on the microscope’s light and move the microscope up and down until neurons are clearly visible. Then mark that position.
    ***Note:*** It is advised to decrease the isoflurane concentration to 1% in the air because neuronal activity decreases during deep anesthesia.
31. Surround the lens with black cement or black manicure to reduce light reflection around the lens (Figure 16).

**Figure 17:**
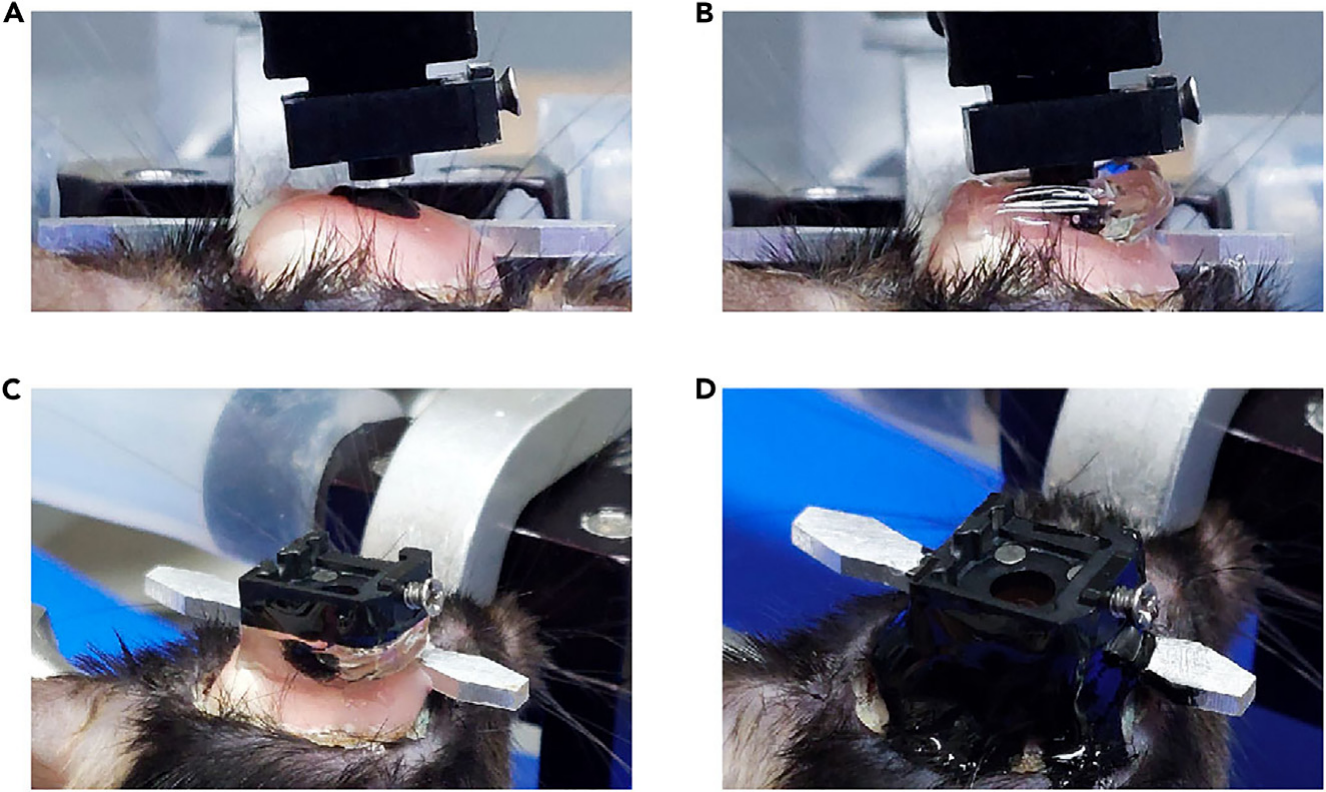
Baseplate installation (A) Align the microscope to capture the largest number of active neurons in the focal plane. (B) Make a foundation for the baseplate. The foundation should not extend beyond the baseplate. (C) Connect the foundation and the baseplate. (D) Cover the foundation with black manicure to prevent light noise.

**CRITICAL:** Make sure the surface of the lens is not covered with black cement or black manicure.

32. Build a foundation for baseplate fixation (Figures 17A–17C). Troubleshooting 4.
  a. Apply UV resin to the baseplate’s underside.
  b. Harden the applied UV resin using a UV beam.
    **CRITICAL:** Make sure the resin does not completely cover the lens surface.
    **CRITICAL:** UV protective eyewear must be worn while using the UV beam to protect the experiment’s eyes.
  c. Repeat steps 32a and b until the resin reaches the height of the baseplate (Figure 17B).
  d. To reinforce the resin foundation, add more resin to the spaces between the baseplate and the resin foundation (Figure 17C).
  e. Verify that the focal plane has not been changed.
    ***Note:*** The focal plane may undergo changes. It is advised that the first focus of the microscope be set in the center of the focus range to accommodate small changes in the focal plane.
33. Use black manicure to conceal the foundation (Figure 17D).
  a. Cover the foundation’s exterior with black manicure to prevent light noise.

***Note:*** Ensure that the light from by the microscope does not escape through the foundation.

34. Cover the baseplate with a cover.

## Expected outcomes

The quality of calcium imaging is influenced by the level of viral expression in the target site, the placement of the lens on the target site, and lens fixation. This protocol provides details on how to control these factors to enable the concurrent recording of more than 1000 neurons in the dorsal CA1 and 200 neurons in the ventral CA1. Dorsal CA1 imaging is more successful (nearly all cases are successful) than ventral CA1 imaging (about half of the cases are successful).

There is a trade-off when choosing the level of expression of calcium indicators: high expression may increase noise signals, while low expression may make it harder to detect calcium signals. We experimented with different viral concentrations and finally settled on a concentration of 2 × 10^12^ vg/mL.

We developed a procedure for securely fixing the lens for dorsal CA1 calcium imaging. We drill a tight hole and remove only a small quantity of the tissue, which permits only a small amount of lens movement. These details are helpful for motion correction, which is a critical step in the analysis of calcium images.

Imaging of a deep brain structure such as the ventral CA1 requires accurate targeting. In this protocol, to decrease targeting errors in the ventral CA1, virus injection and lens implantation are carried out simultaneously. We also use a lens holder that tightly clamps the lens during lens insertion, and lower the lens very slowly during the final stage of lens insertion.

## Limitations

We performed virus injection and lens implantation separately during the dorsal CA1 calcium imaging surgery. This increases the likelihood that the virus expression site differs from the lens implantation site. However, we injected virus at two locations for widespread expression and used a relatively large lens (diameter, 1 mm), increasing the likelihood of targeting dorsal CA1 neurons. The brain tissue above the targeted dorsal CA1 was removed. If excessive tissue is removed, virus-expressing hippocampal tissue may also be removed. Therefore, it is crucial to stop tissue removal as soon as the corpus callosum is discernible, as explained in step 13. In ventral CA1 imaging, precise targeting of the ventral CA1 cell body layer is a challenge because it is located deep in the brain. The success rate was relatively low (50%) despite following the current protocol. We install the baseplate while the mouse is anesthetized. The focal plane may therefore shift between the anesthetized and awake states. Nevertheless, this problem can be resolved simply by refocusing the microscope. Finally, we have not tested an integrated lens which may increase the success rate substantially.

## Troubleshooting

### Problem 1

Neurons are not visible in the field of view.

### Potential solution

First, we recommend waiting ∼8 weeks after virus injection. While waiting, check the field of view. The surgical site might be off the target if the field of view is too dark and the brightness remains constant. Examine the histology, and in future operations, correct the site. If variations in brightness are detectable in the field of view but the shape of the neurons is obscure, adjust the distance between the lens and the microscope, or tilt the microscope. The lens placement is incorrect if the shape of neurons cannot be seen at various microscope distances or angles. Examine the histology, and in future operations, correct the site.

### Problem 2

Black substance appears in the field of view (steps 11, 13, and 23).

### Potential solution

Blood on the lens surface appears as a black substance. Also, scattered black substances may represent inflammation. In either case, you may try to clear the field of view by injecting a ketoprofen solution (100 μL/g; IM) and waiting for a few days. The chance for the black substance to vanish is low, however. Therefore, it is critical to remove blood with saline-soaked Cutanplast during the lens implantation process.

### Problem 3

Brain tissue moves excessively in the field of view (steps 13 and 23).

### Potential solution

Brain tissue can move independently of the skull. Because the lens is secured to the skull rather than the brain, the brain tissue is free to move independently of the lens. You may correct this movement in the captured video using imageJ^3^ with turboreg.^4^ You can also experiment with different surgical details to find the best procedure for minimizing brain movement. For dorsal CA1 imaging, we advise removing a minimal quantity of tissue and lowering the lens more slowly. For ventral CA1 imaging, we advise lowering the lens more slowly and carefully securing the lens before cutting the lens holder.

### Problem 4

Focal plane changes after installing the baseplate (step 32).

### Potential solution

• The pressure in the brain changes as the ear bars' pressure is released, which may the brain with respect to the focal plane. Adjust the focus of the microscope because the shift may be within the microscope’s focal range.
• If you bump the microscope while attaching the baseplate to the foundation the focal plane will change. In this situation, use acetone to carefully remove the foundation and rebuild it.

### Problem 5

Detachment of dental cement from the surgical site (steps 3 and 12).

### Potential solution

• To ensure proper fixation, it is important to perform cementing only when the skull surface is free of debris or moisture. Prior to cementing, ensure thorough drying of the skull and remove any debris using sterilized cotton swabs.
• To ensure adequate support for the cement and lens, it is crucial to securely fasten the screws to the skull. After tightening the screws, gently apply slight pressure with forceps to verify their stability. Add an additional screw if necessary.

## Resource availability

### Lead contact

Further information and requests for resources and reagents should be directed to and will be fulfilled by the lead contact, Min Whan Jung (mwjung@kaist.ac.kr).

### Materials availability

This study did not generate any new unique reagents.

### Data and code availability

This study did not generate any datasets and code.
